# Drug delivery system based on an antibacterial layer-by-layer coating on urinary catheters: an experimental and simulation approach

**DOI:** 10.3389/fbioe.2025.1614509

**Published:** 2025-08-29

**Authors:** Ruth Pulido, Nelson Naveas, Francisco Javier Fernández-Alonso, Miguel Manso-Silván, Leonardo Soriano, Carlos Torres-Ulloa, Karel Mena-Ulecia, Gonzalo Recio-Sánchez, Juan Paulo Garcia-Sandoval, Jacobo Hernández-Montelongo

**Affiliations:** ^1^ Departamento de Química, Universidad de Antofagasta, Antofagasta, Chile; ^2^ Departamento de Física Aplicada, Universidad Autónoma de Madrid, Campus de Cantoblanco, Madrid, Spain; ^3^ Instituto Universitario de Ciencia de Materiales “Nicolás Cabrera” (INC), Universidad Autónoma de Madrid, Campus de Cantoblanco, Madrid, Spain; ^4^ Centro de Microanálisis de Materiales, Universidad Autónoma de Madrid, Campus de Cantoblanco, Madrid, Spain; ^5^ Departmento de Ciencias Matemáticas y Físicas, Universidad Católica de Temuco, Temuco, Chile; ^6^ Departmento de Ciencias Biológicas y Químicas, Universidad Católica de Temuco, Temuco, Chile; ^7^ Facultad de Ingeniería, Arquitectura y Diseño, Universidad San Sebastián, Concepción, Chile; ^8^ Departmento de Ingeniería Química, Universidad de Guadalajara, Guadalajara, Mexico; ^9^ Departamento de Bioingeniería Traslacional, Universidad de Guadalajara, Guadalajara, Mexico

**Keywords:** drug delivery, layer-by-layer (LbL), antibacterial coating, urinary catheters, mathematical model, molecular dynamics

## Abstract

Urinary catheters (UCs) are critical in biomedical applications, but prolonged use increases the risk of catheter-associated urinary tract infections (CAUTIs), a leading cause of healthcare-associated infections (HAIs). The present study presents a dual strategy to create an antibacterial surface on commercial Foley silicone UCs by combining a contact-killing effect with the controlled release of antimicrobial compounds. We designed a drug delivery system using a layer-by-layer (LbL) antibacterial coating of carboxymethylcellulose (CMC) and chitosan-silver (CHI-Ag) complexes, with ciprofloxacin (CFX) as the model drug. The resulting LbL coating, about 1 μm thick, incorporated ${\text{Ag}}^{0}$ and demonstrated a high capacity for CFX loading, releasing over twice the amount ( $70\;\mu g/cm^{2}$ ) compared to uncoated UCs ( $30\;\mu g/cm^{2}$ ). The antibacterial efficacy was significantly higher in the LbL-coated samples, particularly against *S. aureus* compared to *E. coli*. Drug release experiments, modeled using Fick’s second law, indicated a diffusivity of $1.744\times 10^{-5}\;{\text{cm}}^{2}/\text{h}$ . Our mathematical model predicts how variations in drug loading and rest times impact release profiles. Finally, molecular dynamics simulations suggested strong compatibility between CFX and the LbL layers, though with relatively low stability. This dual strategy holds promise for reducing CAUTIs effectively.

## 1 Introduction

Urethral catheters (UCs) are pliable plastic tubes carefully inserted into the urinary bladder through the urethra, serving the purpose of collecting urine in a drainage bag (Chuang and Tambyah, 2021; Newman et al., 2018). As indispensable devices in patient care, catheters rank among the most extensively utilized medical tools in hospital settings. They find application in patients with urinary obstruction, both preceding and following specific surgical procedures. Additionally, catheters play a crucial role in aiding various treatments, such as ensuring precise measurement of urine output, managing incontinence, or facilitating the direct administration of particular medications into the bladder.

Despite their valuable utility, prolonged use of these devices comes with the risk of serious complications, notably the occurrence of catheter-associated urinary tract infections (CAUTIs), recognized as the most prevalent cause of healthcare-associated infections (HAIs) (Teixeira-Santos et al., 2022; Tenke et al., 2017). Alarmingly, approximately 15%–25% of hospitalized patients undergo catheterization, underlining the widespread usage and the associated risks. In both Europe and the United States, the annual incidence of CAUTIs surpasses one million cases, contributing to escalated morbidity and mortality rates (Cai et al., 2023; Öztürk and Murt, 2020).

In this context, rates of CAUTIs are significantly higher in Latin American hospitals than in industrialized countries (Yin et al., 2023). For example, CAUTIs in Chile have shown a notable and consistent increase. Prevalence studies conducted by the Ministry of Health (Unidad de Infecciones Intrahospitalarias, 2020) highlight CAUTIs as the most frequently reported infections across hospitals of varying complexity levels nationwide. Noteworthy is the significant prevalence of CAUTIs observed from 2014 to 2019, primarily attributed to the extensive use of UCs within a substantial patient population. Among the six medical devices examined in the HAIs surveillance report, UCs emerged as the most widely employed, boasting the highest utilization percentage compared to oral parenteral nutrition, other probes, and mechanical ventilation (Unidad de Infecciones Intrahospitalarias, 2020).

In that sense, catheterization could be considered a contraindication as it is closely associated with a heightened probability of CAUTIs (Chuang and Tambyah, 2021). These infections result from the formation of biofilms on UCs, consisting of bacteria that exhibit reduced sensitivity to antibiotics. Additionally, CAUTIs are associated with increased mortality rates, prolonged hospitalization times, and higher hospital expenses (Gray et al., 2023; Hollenbeak and Schilling, 2018). In some cases, this CAUTIs incidence was worsened by the impact of the COVID-19 pandemic due to the large volume of patients requiring advanced medical care and subsequent depleted resources (Hyte et al., 2023; Mitra et al., 2021).

Gram-negative bacteria are the most common causative agents of urinary tract infections (UTIs), including *Escherichia coli*, *Proteus mirabilis*, *Klebsiella pneumoniae*, and *Pseudomonas aeruginosa* (Majumder et al., 2018). Although Gram-positive bacteria are generally less prevalent (Alshomrani et al., 2023), recent studies have reported a growing incidence of *S. aureus* in cases of asymptomatic bacteriuria and complicated UTIs, particularly among elderly individuals, recently hospitalized patients, and those with indwelling urinary catheters (Paudel et al., 2023). Furthermore, *Candida species* can thrive and proliferate in the urinary tract environment (Yao et al., 2022). All these CAUTI-associated pathogens exhibit multiple virulence factors including fimbrial adhesins, urease activity, biofilm formation, and immune evasion proteins. These traits facilitate catheter colonization and persistence. Ciprofloxacin (CFX) targets bacterial DNA gyrase but may be ineffective against sessile, biofilm-embedded cells. Conversely, silver (Ag) exerts membrane-disruptive and metabolic effects independent of active replication, offering complementary protection. Understanding the interplay between drug action and pathogen virulence is key to designing effective coatings (Govindarajan and Kandaswamy, 2022; Sivaramalingam et al., 2024).

In that sense, different strategies have been used to develop antimicrobial surfaces for UCs, which can be grouped into five strategies (Teixeira-Santos et al., 2022): 1) release of antimicrobial compounds (Srisang et al., 2020; Lin et al., 2021; Miao et al., 2023), 2) contact-killing (Gao et al., 2020; Tailly et al., 2021; Bai et al., 2023), 3) anti-adhesive (Yu et al., 2017; Zhang et al., 2019; Prateeksha et al., 2021), 4) disruption of biofilm architecture (Durgadevi et al., 2020; Swidan et al., 2022; da Silva et al., 2024), and 5) benign biofilms to inhibit pathogen colonization (Zhu et al., 2015; Carvalho et al., 2021).

Studies have reported antimicrobial coatings using silver nanoparticles (AgNPs) combined with antibiotics. However, many of these strategies face challenges such as nanoparticle aggregation, burst drug release, and limited control over release kinetics under physiological conditions (Kadirvelu et al., 2024). Our study advances this field by introducing a drug delivery system based on an antibacterial layer-by-layer (LbL) coating applied to commercial silicone urinary catheters (UCs) of two sizes (14Fr and 20Fr). The LbL architecture, fabricated by sequentially assembling carboxymethylcellulose (CMC) with the antibacterial chitosan-silver complex (CHI-Ag), allows modular control of coating thickness, drug loading, and silver integration. CFX was incorporated as a model drug to investigate controlled release performance. This dual-action system combines contact-killing properties with the sustained release of antimicrobial compounds. The coated UCs were subjected to morphological and physicochemical characterization, and their antibacterial efficacy was evaluated against both gram-negative *E. coli* and gram-positive *S. aureus* strains. Biological *in vitro* assays were performed using pristine, coated, and CFX-loaded coated samples. To elucidate the mechanisms of CFX loading and release, we implemented a mathematical model based on unidirectional diffusion governed by Fick’s second law. Additionally, molecular dynamics (MD) simulations were employed to investigate the interactions between CFX and the coating matrix, using catheter-relevant geometries (14Fr/20Fr) to better represent real-world performance.

## 2 Materials and methods

### 2.1 Materials

14Fr and 20Fr Foley silicone UCs were acquired from Chen Kang^®^ (China); their nominal diameters were 4.7 mm and 6.7 mm, respectively. The following reagents were procured from Sigma–Aldrich (United States): polyethylenimine (PEI), a 50 wt% solution in water with an average molecular weight (Mw) $\approx 7.5\times 10^{5}$ g/mol (PDI ≤ 1.3) determined using light scattering; carboxymethylcellulose (CMC) with an average Mw ≈9× 10^4^ g/mol and a degree of substitution of 0.9; chitosan (CHI) with an average Mw ≈5× 10^4^ g/mol and 75%–85% deacetylated; methylene blue (MB) with Mw = 373.90 g/mol; and Rose Bengal (RB) with Mw = 1,017.64 g/mol.

Additionally, the following chemicals and substances were obtained from Merck (Germany): silver nitrate ( ${AgNO}_{3}$ ) with Mw = 169.87 g/mol; glacial acetic acid ( ${CH}_{3}$ COOH) with Mw = 60.05 g/mol; ascorbic acid ( $C_{6}$ H_8_O_6_) with Mw = 176.12 g/mol; hydrochloric acid (HCl) with Mw = 36.46 g/mol; sodium hydroxide (NaOH) with Mw = 40.01 g/mol; and hydrochlorinated ciprofloxacin (CFX) ( $C_{17}$ H_18_FN_3_O_3_ ⋅ HCl ⋅ $H_{2}$ O) with Mw = 385.82 g/mol. Milli-Q water was also obtained from Merck (Germany).

### 2.2 Layer-by-layer coating

UCs samples were cut to a length of 2.5 cm using a scalpel and sealed at both ends using a silicone gun. Subsequently, the samples underwent oxidation in an oxygen plasma equipment (Harrick Plasma, United States) under low pressure (0.2 mmHg) for 15 min. This process enhances surface hydrophilicity and activates silanol groups on the PDMS surface, which improves the subsequent adsorption of PEI and the adhesion of the LbL assembly (Hernandez-Montelongo et al., 2017). Following this, a preliminary layer of PEI solution (1 mg/mL, pH = 4) was applied for 15 min, followed by rinsing with Milli-Q water, pre-adjusted to pH 4.

The LbL coating incorporating silver was then executed using the method proposed by (Naveas et al., 2023). Briefly, the samples were immersed for 10 min in a CMC solution (0.1% m/v, pH = 4) and rinsed with Milli-Q water adjusted to pH 4. Subsequently, the samples were immersed for 10 min in a chitosan-silver (CHI-Ag) solution (0.1% m/v CHI solution with 0.1 mM ${AgNO}_{3}$ , pH 4) and then rinsed with Milli-Q water at the same pH. This cycle was repeated ten times. Following the coating process, the samples were immersed in ascorbic acid for 3 h to reduce the silver nitrate to silver. Finally, the samples were thoroughly rinsed with Milli-Q water adjusted to pH4, the silicone seals were removed, and the samples were dried.

### 2.3 Physicochemical characterization

The surface wettability of the samples was assessed using a water contact angle measuring system (Dropletlab, Canada) in static sessile drop mode. A 10 μ L water drop was employed for each measurement, and five measurements were conducted. Roughness measurements were performed using a Dektak 150 stylus profilometer (Veeco, United States) with a force of 1.0 mg and a scan speed of 17 μ m/s.

For the evaluation of carboxylic and amino groups in UCs with LbL coating, samples underwent immersion in MB (0.001 M, pH = 7) and RB (0.001 M, pH = 5) solutions. This was followed by rinses with Milli-Q water. Subsequently, stains from the samples were extracted for UV–vis spectroscopy measurements in absorbance mode. Samples treated with MB were immersed in a 5% (v/v) solution of glacial acetic acid, while those with RB were immersed in a 0.1 M NaOH solution. The absorbance values for MB and RB were measured at 663 nm (Eltaweil et al., 2020) and 545 nm (Bayraktutan, 2020), respectively, using a UV-Vis spectrophotometer (Evolution 220 model, Thermo Scientific, United States).

Chemical analysis of the samples was conducted using Attenuated Total Reflectance Fourier-Transform Infrared Spectroscopy (ATR-FTIR). An FTIR spectrometer coupled with an ATR accessory using a zinc selenide crystal (CARY 630 FTIR Agilent Technologies, United States) was employed within the range of 4,000 to 600 ${cm}^{-1}$ with a resolution of 1 ${cm}^{-1}$ (NS = 4). The obtained spectra underwent mathematical processing through data smoothing and normalization.

The morphology of the samples was explored using a variable pressure scanning electron microscope (VP-SEM, SU-3500 Hitachi, Japan) with an acceleration voltage of 10 kV. The acquired images underwent processing using the freely available ImageJ software, version 1.52k. For elemental mapping and atomic percentage determination, energy-dispersive X-ray analysis (EDX) was employed with an INCA X-sight system from Oxford Instruments integrated into the VP-SEM equipment.

X-ray Photoelectron Spectroscopy (XPS) was employed to analyze the surface chemical composition of the samples. XPS spectra were obtained using the Surface Analysis Station 150 XPS RQ300/2 (TAIB Instruments) equipped with a hemispherical electron analyzer and a Mg-anode X-ray source. The pass energy was set at 20 eV, providing an overall resolution of 0.9 eV. All XPS binding energies were referenced to the adventitious C 1s carbon peak at a binding energy of 284.6 eV to account for surface charging effects. The XPS spectra were fitted using the CasaXPS software.

In-depth profiling of the silver into LbL coating was studied through Rutherford Backscattering Spectroscopy (RBS). RBS experiments were conducted at the standard beamline of the Center of Micro-analysis of Materials (CMAM, Spain) (Redondo-Cubero et al., 2021), which houses a 5 MV Cockroft-Walton tandetron accelerator. In these experiments, 4 MeV He 2+ ions were utilized, and the scattered ions were detected at a scattering angle of 170° using a Si semiconductor particle detector. The vacuum conditions were maintained at approximately 5 × 10 −5 Pa. Simulations and spectrum fitting were performed using SIMNRA 7.02 software (Mayer, 2014).

### 2.4 Drug loading and release profiles

Following the assembly of the LbL coating, each sample underwent a 12-h immersion in a ciprofloxacin (CFX) solution with a concentration of 1.33 mg/mL. Subsequently, the samples were thoroughly rinsed with Milli-Q water and allowed to dry. To generate the release profiles of the CFX-loaded samples, they were placed in a phosphate-buffered saline (PBS) solution within a horizontal shaker set at 37 °C, pH of 7.6, and 50 rpm. The concentration of CFX in PBS was determined at various release times over a 15-day period using a UV-Vis spectrophotometer (Evolution 220 model, Thermo Scientific, United States). CFX was detected at a wavelength of 275 nm (Uddin et al., 2022). All experiments were conducted in triplicate, with non-functionalized samples serving as controls in the kinetic release experiments.

### 2.5 Antibacterial assays

The antibacterial activity of the samples was assessed using the agar diffusion method against two bacterial strains: *Escherichia coli* ATCC 25922 (gram-negative) and *Staphylococcus aureus* ATCC 25923 (gram-positive). Mueller-Hilton agar served as the medium for the diffusion method test. Bacterial strains were adjusted to a 0.5 McFarland standard concentration and inoculated onto solid media under aseptic conditions. Samples were then applied vertically onto the surface of the solid media and allowed to dry for 1 h. The inoculated plates were incubated for 24 h at 35 °C, after which the inhibition zones around the samples were measured from photographs of the plates using ImageJ software. Data were analyzed statistically by analysis of variance (ANOVA) with a subsequent Tukey post-hoc test using OriginLab v. 2022b software; p-values of 0.05 or less were considered statistically significant. All assays were performed in triplicate.

### 2.6 Mathematical model

The mathematical model utilized in this study is built around three consecutive processes centered on unidirectional diffusion governed by Fick’s second law (Hernandez-Montelongo et al., 2022). These processes include drug loading, where samples are loaded with a highly concentrated drug solution; a resting stage, during which the drug continues to diffuse into the matrix samples; and drug delivery, where the drug is released from the samples into a PBS solution at 37 °C, simulating bodily fluid conditions.

**FIGURE 1:**
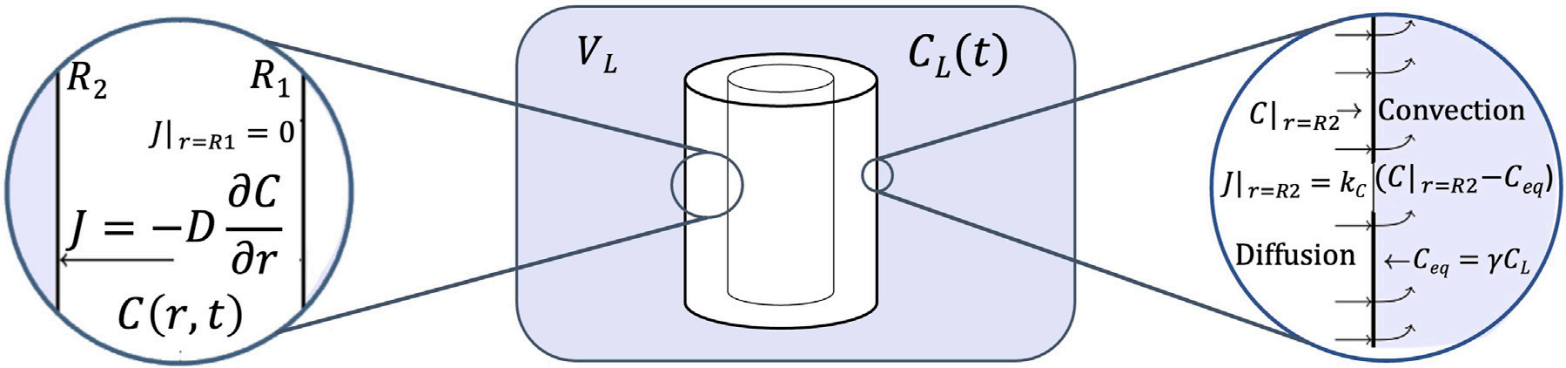
Scheme of the drug delivery system.

The diffusion equation for the delivery system is shown in (Equation 1):

$$\frac{\partial C\left(r,t\right)}{\partial t}=D\left(\frac{\partial^{2}C\left(r,t\right)}{\partial r^{2}}+\frac{1}{r}\frac{\partial C\left(r,t\right)}{\partial r}\right),\;\begin{array}{c} R_{1}<r<R_{2}\\ t>0 \end{array}$$ (1)

where C is the CFX concentration in the UC, which depends on time, t , and radial position, r , D represents the diffusivity of CFX in the silicone urinary catheter (UC), which is assumed to remain constant. The reason for this is that, despite the structural differences between pristine silicone and the LbL coating, diffusivity (D) was assumed constant due to the significantly smaller thickness ( ∼ 1 μm ) of the LbL film relative to the catheter wall (1 mm ). As such, the LbL contribution to the overall diffusion pathway is minimal. Partition coefficients were used to account for differences in affinity. Finally, $R_{1}$ and $R_{2}$ are the inner and outer diameters, respectively (Figure 1).

At the beginning, the UC does not contain CFX, therefore the initial condition must be Equation 2:

$$C\left(r,0\right)=0,\;R_{1}<r<R_{2}$$ (2)

The inner region of the UC only contains air because it is closed before the experiment starts, thus we assume that CFX does not diffuse to this region and the boundary condition at $r=R_{1}$ is (Equation 3):

$$\frac{\partial C\left(R_{1},t\right)}{\partial r}=0,$$ (3)

On the other hand, the CFX’s flux on the external surface of the UC varies depending on the stage of the process. At the CFX load stage, from t=0 to $t=t_{0}$ , due to the use of a highly concentrated solution of CFX, the external CFX concentration is practically constant and equal to $C_{L}$ (CFX concentration in the liquid bulk) and a convective process takes place. Thus, the boundary condition at $r=R_{2}$ is assumed to be:

$$-D\frac{\partial C\left(R_{2},t\right)}{\partial r}=k_{L}\left[C\left(R_{2},t\right)-\gamma_{L}C_{L}\right],\;0<t\leq t_{0}$$ (4)

Here in (Equation 4), $k_{L}$ and $\gamma_{L}$ are the convective and equilibrium partition coefficients, respectively, between the UC surface and the CFX solution. Here we assume that $k_{L}$ and $\gamma_{L}$ may be different for the non-coated and coated UC. Then, at the rest stage from $t=t_{0}$ to $t=t_{1}$ , the external surface of the UC is in contact with air, thus we assume that CFX does not diffuse to this region and the boundary condition at $r=R_{2}$ is (Equation 5):

$$\frac{\partial C\left(R_{2},t\right)}{\partial r}=0,\;t_{0}<t\leq t_{1}.$$ (5)

Finally, at the delivery stage, the UC is in contact with the PBS solution that initially has zero CFX concentration, but as the CFX diffuses from the UC surface to the PBS solution, the external CFX concentration in the PBS solution, $C_{D}\left(t\right)$ , increases. Thus, the boundary condition at $r=R_{2}$ is assumed to be (Equation 6):

$$-D\frac{\partial C\left(R_{2},t\right)}{\partial r}=k_{D}\left[C\left(R_{2},t\right)-\gamma_{D}C_{D}\left(t\right)\right],\;t>t_{1}.$$ (6)

while the external CFX concentration in the PSB solution varies according to (Equation 7):

$$\begin{array}{c} \frac{dC_{D}\left(t\right)}{dt}=\frac{2\pi R_{2}L}{V_{\mathrm{PBS}}}k_{D}\left[C\left(R_{2},t\right)-\gamma_{D}C_{D}\left(t\right)\right],\\ \;t>t_{1},\text{and}\;C_{D}\left(t_{1}\right)=0. \end{array}$$ (7)

where $2\pi R_{2}L$ is the UC external surface area, $V_{\mathrm{PBS}}$ is the volume of the PBS solution, while $k_{D}$ and $\gamma_{D}$ are the convective and equilibrium partition coefficients, respectively, between the UC surface and the PBS solution. As in the case of the loading stage, we assume that $k_{D}$ and $\gamma_{D}$ may be different for the non-functionalized and functionalized UC. Here, we assume that the PSB solution has an homogeneous concentration, $C_{D}\left(t\right)$ , because it is perfectly mixed.

In particular, if we consider a well mixed convection during the loading and delivery stages, then $k_{L}\gg D$ and $k_{D}\gg D$ , therefore boundary conditions (4) and (6) can be reduced to (Equations 8, 9):

$$C\left(R_{2},t\right)=\gamma_{L}C_{L},\;0<t\leq t_{0}$$ (8)

$$C\left(R_{2},t\right)=\gamma_{D}C_{D}\left(t\right),\;t>t_{1}$$ (9)

while the dynamical behavior of the external CFX concentration in the PBS solution becomes (Equation 10):

$$\frac{dC_{D}\left(t\right)}{dt}=-\frac{2\pi R_{2}L}{V_{\mathrm{PBS}}}D\frac{\partial C\left(R_{2},t\right)}{\partial r},\;t>t_{1},\;\text{and}\;C_{D}\left(t_{1}\right)=0.$$ (10)

In summary, the model is described by PDE (Equation 1), initial condition (Equation 2), and boundary condition (Equation 3) for all the domain of time. The boundary conditions (Equation 8) and (Equation 5) are used at load and rest stages, respectively, while boundary condition (Equation 9) and ODE (Equation 10) are used at delivery stage.

### 2.7 Molecular dynamics (MD) simulations

To gain deeper insights into the mechanisms governing the experimental behavior of controlled CFX release, quantum mechanical tools were employed in this study (Aazam and Thomas, 2024). The chemical structure of CFX was retrieved from the DrugBank database, identified by the code DB00537 (Knox et al., 2024). PDMS, CMC and the CHI-Ag complex were sketched using the freely available online tool Marvin JS (https://marvinjs-demo.chemaxon.com/latest/demo.html). These structures were obtained in XYZ format and individually optimized to their minimum energy states using the valence triple-zeta with two sets of polarization functions (def2-TZVPP) basis set for all atoms and the Becke, 3-parameter, Lee–Yang–Parr (B3LYP) hybrid functional (Weigend and Ahlrichs, 2005; Becke, 1992; AL-Khaykanee, 2013; Orio et al., 2009), implemented in the Orca 5.0.1 software package (Neese, 2012; Neese, 2022). The full optimized geometry of all molecules was verified by examining their imaginary frequencies.

After confirming that all molecules had reached their energy minima, the systems under investigation were assembled. These systems comprised two complexes: complex-1 consisted of three layers, with the inner and outer layers composed of PDMS molecules and a layer of three CFX molecules sandwiched between them at a distance of 3Å. Complex-2 consisted of six layers: the outermost layer contained two CFX molecules, followed by a layer of CHI-Ag at a distance of 3Å, a layer of two CFX molecules, a layer of CMC, another layer of two CFX molecules, a layer of one CHI-Ag molecule, and an innermost layer of three CFX molecules. Both complexes were constructed using the Chemcraft version 1.8 program for Linux (Andrienko, 2010) and simulated using the *ab initio* molecular dynamics (AIMD) method (Bocharov et al., 2020; Tse, 2002; Marx and Hutter, 2000). Each system underwent a simulation period of 100 fs (2000 steps) with a time step of 0.5 fs at a temperature of 298.15 K. All *ab initio* MD simulations were conducted using the Orca 5.0.1 program package (Neese, 2012; Neese, 2022; Snyder and Kucukkal, 2021).

## 3 Results and discussion

**FIGURE 2:**
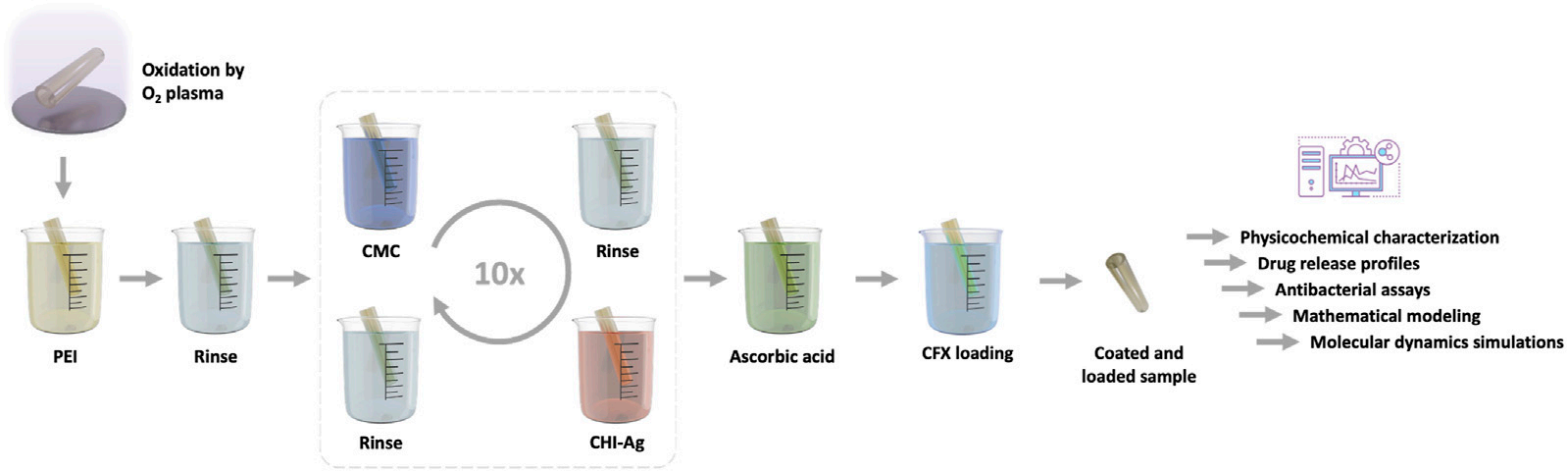
Schematic representation illustrating the assembly process of the coating composed of carboxymethylcellulose (CMC) and chitosan-silver (CHI-Ag) leading to a the Layer-by-Layer technique Ag loaded coating (LbL): The sample is immersed alternately in the respective polysaccharide electrolyte, followed by rinsing steps and ${\text{Ag}}^{+}$ ion reduction to ${\text{Ag}}^{0}$ by ascorbic acid.

In this work, UCs of varying diameters (14Fr and 20Fr) underwent a coating process involving a thin film of CMC/CHI-Ag, assembled through a layer-by-layer technique (LbL). The synthesis process is outlined schematically in Figure 2. Given that polydimethylsiloxane (PDMS) serves as the basis for the silicone UCs (Zare et al., 2021), the surface of these devices is inherently hydrophobic. Thus, prior to coat the samples, a surface pretreatment involving $O_{2}$ plasma oxidation, succeeded by PEI grafting was conducted. This well-established strategy ensures a more uniform and controlled synthesis of LbL films (Hernandez-Montelongo et al., 2017). Subsequently, a ten-cycles assembly process was executed, culminating in immersion in an ascorbic acid solution to facilitate the reduction of ${\text{Ag}}^{+}$ ions to ${\text{Ag}}^{0}$ within the coating matrix (Naveas et al., 2023).

**FIGURE 3:**
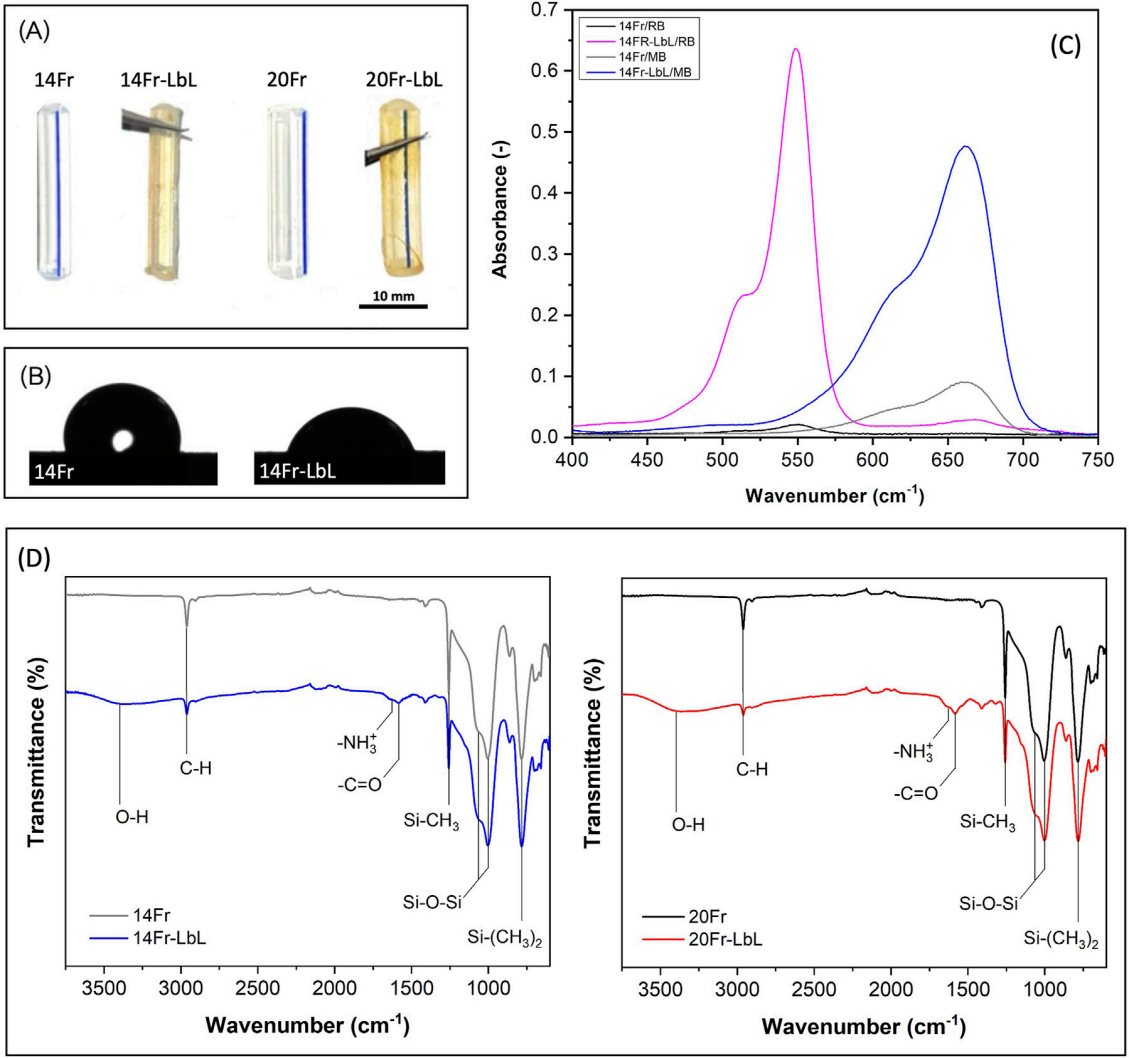
**(A)** Urinary catheters before and after the layer-by-layer coating. **(B)** Water contact angle of pristine and coated sample (14Fr). **(C)** Absorbance of Rose Bengal and Methylene Blue obtained from pristine and coated samples (14Fr). **(D)** ATR-FTIR spectra of urinary catheters before and after the layer-by-layer coating.

Figure 3A depicts UCs before and after the LbL coating, evident from the light brown appearance. Following the LbL treatment, the wettability of UC samples transitioned to a hydrophilic behavior, registering a change from 100 ± 2° to 69 ± 5° for the 14Fr catheter and from 103 ± 2° to 68 ± 7° for the 20Fr catheter. This shift is attributed to the presence of carboxylic groups in CMC, ammonium groups in CHI, and Ag in the coating. In Figure 3B, contact angle images of the 14Fr (hydrophobic) and 14Fr-LbL (hydrophilic) samples are presented. Concurrently, the roughness experienced a significant alteration due to the LbL coating. Both 14Fr and 20Fr samples saw a substantial increase in roughness. 14Fr nearly doubled its roughness from 0.46 ± 0.13 μ m to 0.93 ± 0.33 μ m, while 20Fr increased from 0.70 ± 0.22 μ m to 1.18 ± 0.42 μ m. These values indicate the formation of a micro-structured coating on the surface.

UV–Vis spectroscopy served as a tool to indirectly identify the carboxylic groups of CMC (negatively charged) by utilizing the oppositely charged stain methylene blue (MB) (positively charged) (Eltaweil et al., 2020). Likewise, to indirectly detect the ammonium groups of CHI (positively charged), the stain Rose Bengal (RB) (negatively charged) was applied (Bayraktutan, 2020). Figure 3C illustrates the absorbance peaks of MB and RB for both the 14Fr and 14Fr-LbL samples. The pristine 14Fr sample displayed low absorbance values: 0.089 for MB and 0.020 for RB. In contrast, the coated 14Fr-LbL sample exhibited significantly higher absorbance peaks, measuring 0.476 for MB and 0.635 for RB. A similar trend was observed in the 20Fr and 20Fr-LbL samples.

The chemical analysis of the samples was conducted using ATR-FTIR to directly identify the surface modifications (Figure 3D). The bending vibrations of the fingerprint functional groups of PDMS were discerned, including Si-( ${CH}_{3}$ )_2_ (786 ${cm}^{-1}$ ), symmetric bending of Si-CH_3_ (1,263 ${cm}^{-1}$ ), stretching of CH (2,960 ${cm}^{-1}$ ) from CH_3_, and Si-O-Si stretching at 1,065 ${cm}^{-1}$ and 1,007 ${cm}^{-1}$ , observed in the spectra of both control samples (14Fr and 20Fr) and coated samples (14Fr-LbL and 20Fr-LbL) (Bodas and Khan-Malek, 2006). Furthermore, a broad band at 3,400 ${cm}^{-1}$ corresponding to O-H stretching of CMC and CHI was detected. ${{NH}_{3}}^{+}$ bending vibration at 1,624 ${cm}^{-1}$ of CHI and -C=O antisymmetric stretching vibration at 1,580 ${cm}^{-1}$ of CMC were also identified (Bozoğlan et al., 2020).

**FIGURE 4:**
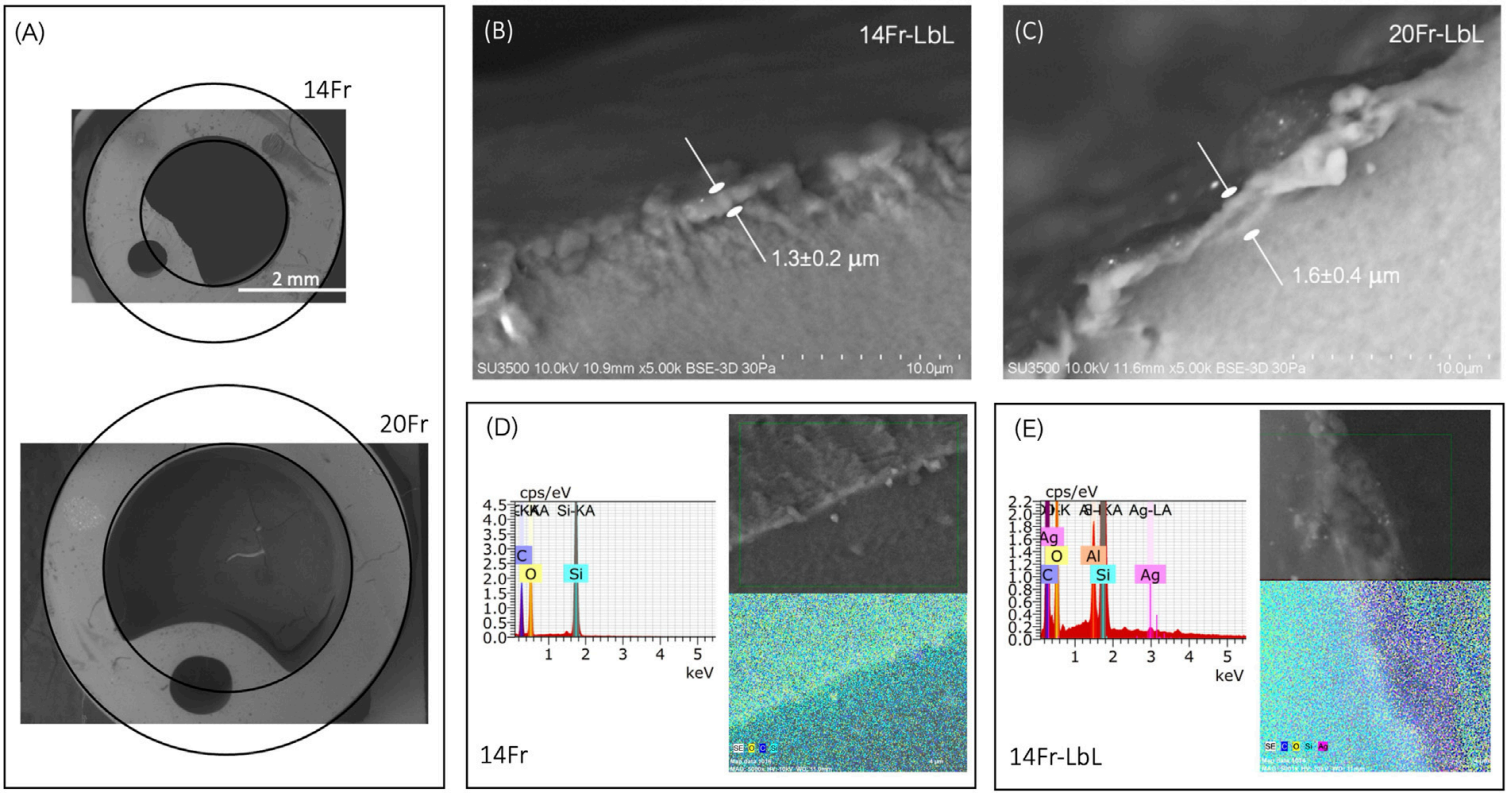
**(A)** Cross-sectional SEM images of 14Fr and 20Fr catheters. **(B)** Thickness of the 14Fr-LbL film. **(C)** Thickness of the 20Fr-LbL film. **(D)** EDX analysis and mapping of the 14Fr sample. **(E)** EDX analysis and mapping of the 14Fr-LbL sample.

Figure 4A displays cross-sectional SEM images of both UCs. The 14Fr sample exhibited external and internal diameters of 4.7 mm and 2.7 mm, respectively. In the case of the 20Fr sample, its external and internal diameters were 6.7 mm and 4.7 mm, respectively. Both UCs presented a wall thickness of 1 mm. Moreover, the typical inflation cuff orifice of Foley catheters was also observed in both samples. UCs submitted to the layer-by-layer treatment showed a thin coating of 1.3 ± 0.2 μ m for 14Fr (Figure 4B) and 1.6 ± 0.4 μ m for 20Fr (Figure 4C). To confirm that the observed coating belongs to the LbL film, EDX analysis was performed on samples. In the pristine 14Fr catheter, C, O, and Si were detected, in agreement with their PDMS composition (Figure 4D). EDX mapping also presents these elements on the edge of the surface of the sample. However, in the case of the coated sample (14Fr‐LbL; Figure 4E), Ag was also detected at a mass percent of 1.5 ± 0.6%. EDX mapping shows a high signal of Ag in the coating matrix. For 20Fr-LbL, a similar tendency was observed with a mass percent of 1.6 ± 0.2%.

**FIGURE 5:**
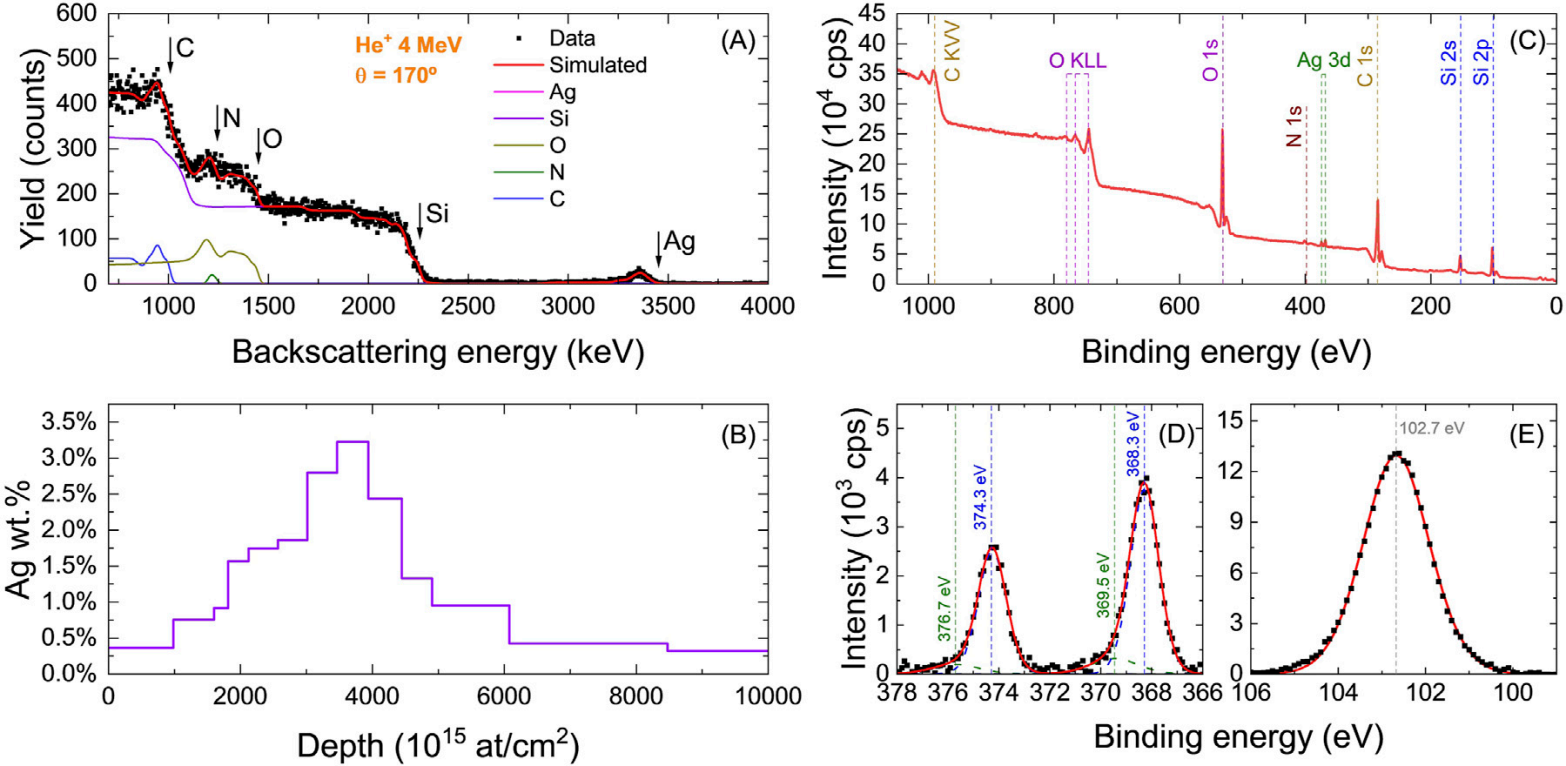
**(A)** RBS experimental and simulated spectra and **(B)** derived in-depth concentration profile of Ag obtained via RBS fitting. XPS analysis on 14Fr-(LbL-Ag) sample: **(C)** survey spectra of 14Fr-(LbL-Ag) sample and, XPS spectra of **(D)** Ag 3d and **(E)** Si 2p core levels.

Rutherford Backscattering Spectrometry (RBS) was performed to analyze the diffusion of silver (Ag) in the UCs. Figure 5A shows the RBS spectrum obtained for the 14Fr-LbL sample. Five element-related signals can be observed, corresponding to carbon (C), nitrogen (N), oxygen (O), silicon (Si), and silver (Ag). The Ag signal shows a peak at a backscattering energy of 3,359 keV, whereas, for Ag atoms on the surface, a backscattering energy of 3,447 keV would be expected. This indicates that silver has infiltrated towards the bulk material of the LbL coating.

The in-depth compositional profile of silver was estimated by fitting the RBS spectrum using the Simnra 7.03 software, as shown in Figure 5B. For this analysis, a structure composed of up to 20 layers of material was considered, each with varying thickness and concentrations of C, N, O, Si, and Ag. The results confirm that the concentration of Ag on the coating surface is only 0.4%, while deeper within the material, the weight percentage increases up to 3.2%.

The surface chemical composition of the UCs was studied via X-ray Photoelectron Spectroscopy (XPS). Figure 5C shows the XPS spectrum of the 14Fr-LbL samples. The survey spectrum reveals the presence of carbon (C), nitrogen (N), oxygen (O), silicon (Si), and silver (Ag). A quantitative estimation of the surface composition was performed based on the intensity of the signals associated to each element, using the sensitivity factors compiled by (Wagner et al., 1981). In this way, the surface concentration of silver was estimated to be 0.4%, consistent with the amount of silver observed in the outermost layer as estimated by RBS.

Figure 5D shows the spectral region corresponding to Ag 3d. Two peaks are observed at 368.3 eV and 374.3 eV, corresponding to the photoemission peaks associated with the Ag $3d_{5/2}$ and $3d_{3/2}$ , respectively. The binding energy of these peaks is consistent with ${\text{Ag}}^{0}$ (Ferraria et al., 2012; Firet et al., 2019). In addition to the metallic peaks, two additional bands were detected at 369.5 eV and 376.7 eV. Previous studies have attributed these bands to silver clusters smaller than 4 nm (Calderon V. et al., 2013). However, the intensity of these bands is relatively low, accounting for approximately 15% of the total intensity of the peak associated to silver.

Figure 5E shows the spectral region corresponding to Si 2p. The region is accurately fitted with a single Gaussian-Lorentzian peak centered at 102.7 eV, which is consistent with the formation of Si-O bond (Kaur et al., 2016; Kong et al., 2020).

**FIGURE 6:**
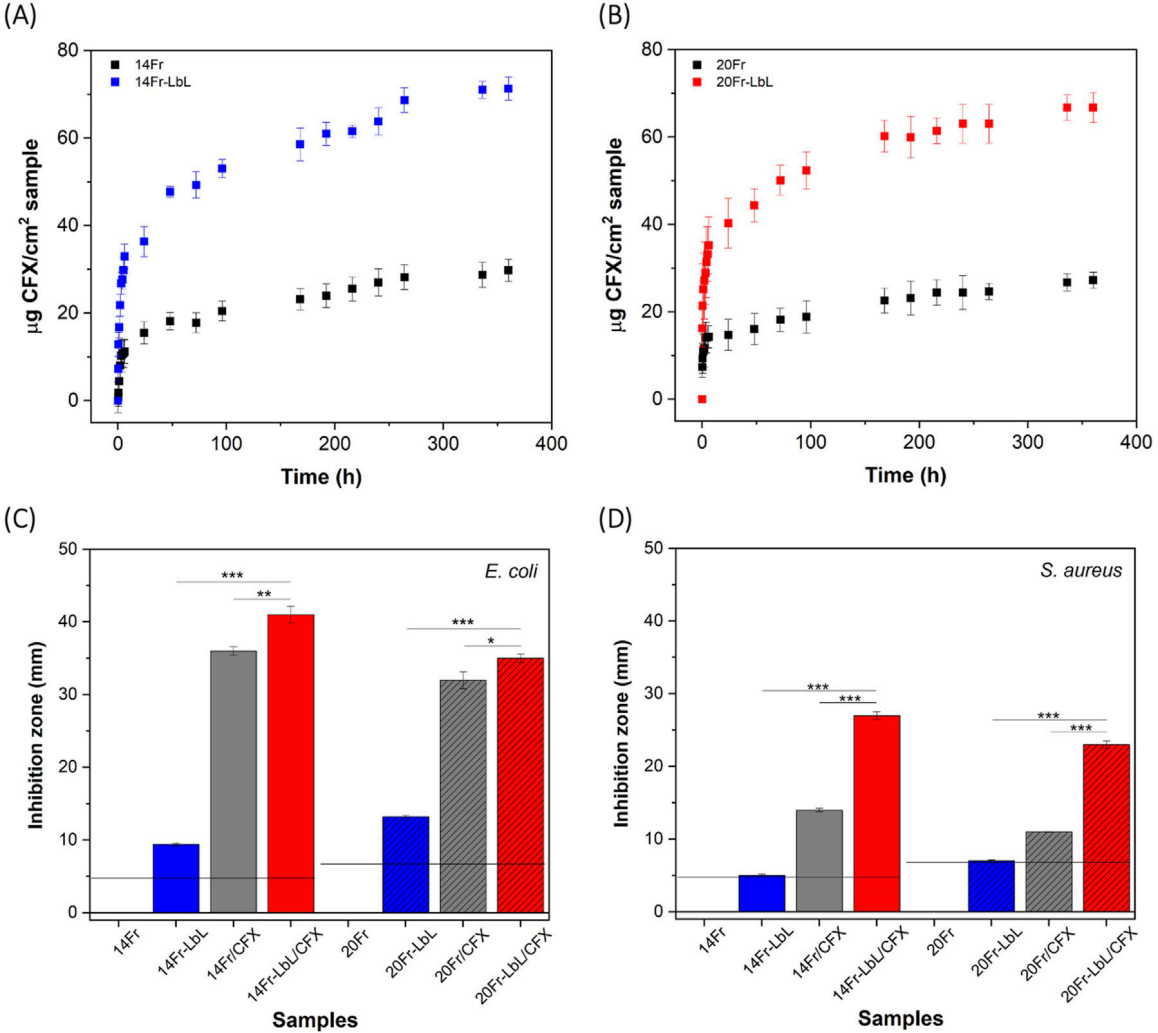
Drug release profiles of CFX from: **(A)** 14Fr and 14Fr-LbL, and **(B)** 20Fr and 20Fr-LbL. Antibacterial tests of samples against: **(C)** *Escherichia coli*, and **(D)** *Staphylococcus aureus*. Labels mean 14Fr and 20Fr are uncoated, 14Fr-LbL and 20Fr-LbL are LbL coated, 14Fr/CFX and 20Fr/CFX are uncoated but CFX-loaded, 14Fr-LbL/CFX and 20Fr-LbL/CFX are LbL-coated and CFX-loaded. Results represent mean ± SD of three measurements, statistically interpreted by analysis of variance (ANOVA) with subsequent Tukey post-hoc test. Significant differences are presented as * is p-value ≤ 0.05, ** is p-value ≤ 0.01 and *** is p-value ≤ 0.001. Horizontal lines indicate the diameter of UCs.

To evaluate the ciprofloxacin (CFX) delivery performance of the coated samples, experiments were conducted in PBS at 37 °C and pH 7.6 under stirring conditions. A pH of 7.6 is close to physiological conditions and falls within the typical range of urine (pH 4.5–8.0), which can vary depending on hydration levels and overall health status. Both control and coated samples were loaded with highly concentrated solutions of the antibiotic for 12 h and then stored for 24 h (resting stage) prior to use in the experiments. The drug release profiles obtained are presented in Figures 6A,B. The results indicate that pristine samples (14Fr and 20Fr) exhibited significant release of CFX, demonstrating the chemical compatibility between the antibiotic’s structure and the silicone substrates (Hui et al., 2008). In that sense, the released amount of CFX for both control samples was similar, approximately 30 μ g/cm^2^. On the other hand, the coated samples demonstrated a substantial capacity to release higher amounts of CFX, despite having only a micron-thick LbL film on both UCs. Both coated samples, 14Fr-LbL and 20Fr-LbL, released around 70 μ g/cm^2^, more than double the amount released by the control samples. This illustrates the high capacity of the LbL matrix to load and release CFX. Although the long-term durability of the LbL coating under storage conditions was not tested in this study, similar polyelectrolyte-based systems have shown stability over months when stored in dry, dark environments. Future work will evaluate performance degradation over time.

Furthermore, Figures 6C,D illustrate the antibacterial efficacy of the samples against *E. coli* and *S. aureus*. For the *E. coli* strain (Figure 6C), the pristine samples (14Fr and 20Fr) exhibited no antibacterial activity. However, the coated samples 14Fr-LbL and 20Fr-LbL displayed inhibition areas of 10 and 13 mm, respectively. These inhibition zones noted for the LbL-only samples might indicate limited diffusion of silver ions caused by surface-anchored ${\text{Ag}}^{0}$ because *E. coli*, and *S. aureus*, are generally silver-tolerant up to 1.7–54 mg/L (Gugala et al., 2019; Hochvaldová et al., 2024) by involving the aggregation of AgNPs leading to the formation of black precipitates: *E. coli* triggers flagellin-mediated AgNPs aggregation, whereas *S. aureus* accelerates biofilm-induced aggregation (Hochvaldová et al., 2024). Our observation of growth inhibition suggests that the local ${\text{Ag}}^{+}$ concentration at the catheter surface reached, at least transiently, in the 0.05–0.20 ppm (5–20 μ g/L) range since these concentrations fall within the bactericidal window reported for silver-polymer coatings (Jung et al., 2008; Aldakheel et al., 2023). On the other hand, the stronger antibacterial effect observed in the *E. coli* assay compared to *S. aureus* can be attributed to the greater efficacy of silver ions against Gram-negative bacteria. This is likely due to the thicker peptidoglycan layer in *S. aureus*, which may hinder the penetration of silver ions through the bacterial cell wall (Jung et al., 2008). Moreover, due to the loading and release capacity of the control samples for CFX, they exhibited inhibition zones of 35 mm for 14Fr/CFX and 32 mm for 20Fr/CFX. In the case of coated samples loaded with CFX, its enhanced antibacterial activity is chiefly attributable to increased CFX loading and diffusion: 14Fr-LbL/CFX and 20Fr-LbL/CFX achieved inhibition zones of 40 and 35 mm, respectively. It is noteworthy that samples loaded with CFX, both uncoated and coated, exhibited similar inhibition diameters because they released similar amounts of CFX. It is worth noting that both LbL and non-LbL CFX-loaded catheters exceeded the CLSI-defined inhibition zone threshold of ≥ 26 mm for *E. coli* susceptibility to CFX (Humphries et al., 2019) suggesting that both approaches deliver clinically meaningful antibiotic levels.

Also, it is important to note that an increase in the surface roughness and the transition from a hydrophobic to a hydrophilic surface (as shown in Figures 3A,B) after the LBL procedure may enhance bacterial adhesion. However, this effect could be counteracted by the strong antibacterial properties of silver and CFX, which help prevent early-stage colonization. Nonetheless, further studies are needed to evaluate long-term biofilm formation under dynamic conditions in the urinary tract.

In the case of the *S. aureus* strain (Figure 6D), pristine samples also showed no antibacterial effect, while coated samples only exhibited an inhibition zone corresponding to the diameter of the UCs: 4.7 mm for 14Fr-LbL and 6.7 mm for 20Fr-LbL. This indicates that the LbL coating solely functioned as an antibacterial surface. However, samples loaded with CFX demonstrated increased antibacterial efficacy: 14Fr/CFX and 20Fr/CFX exhibited inhibition zones of 15 and 10 mm, respectively.Notably, the combined antibacterial activity resulting from the combination of the LbL coating and the CFX antibiotic was significantly enhanced for this strain. The 14Fr-LbL/CFX and 20Fr-LbL/CFX samples demonstrated inhibition zones of 27 mm and 23 mm, respectively. Both values exceed the CLSI threshold of ≥ 21 mm for *S. aureus* susceptibility to CFX (Din et al., 2022), supporting the potential clinical utility of these coatings, even though *S. aureus* is not typically a predominant uropathogen. While these results against *E. coli* and *S. aureus* are promising, it is important to note that in clinical settings, catheter surfaces are often subject to biofilm formation and encrustation—conditions that can impair antibiotic diffusion and silver efficacy. Biofilm formation and catheter encrustation, often exacerbated by urease-producing bacteria and alkaline urine, can form diffusion barriers that reduce antibiotic release and surface contact activity. Although our dual-action coating may delay biofilm development, future studies should assess release profiles under encrusted or biofilm-laden conditions to more accurately evaluate its performance and long-term clinical relevance.

To gain deeper insights into the mechanisms governing the release of CFX from both uncoated and coated samples, we developed a mathematical model accounting for the drug loading step and the resting stage (Section 2.6). The kinetic parameters obtained from this model are presented in Table 1. Given that the wall thickness of the UCs is 1 mm and the LbL coating is a micron-thick film, we assumed that the diffusivity ( D ) remains constant for both uncoated and coated samples at $1.744\times 10^{-5}$ cm^2^/h. However, the partition rates ( γ ) listed in Table 1, representing the drug solid–liquid partition coefficient associated with equilibrium, varied depending on the type of solution used: highly concentrated CFX for loading ( $\gamma_{L}$ ) or the PBS solution used for drug delivery experiments ( $\gamma_{D}$ ), and the type of UC sample (uncoated or coated). Notably, the values for coated UCs were higher than those for the uncoated controls, indicating a greater affinity of CFX for the LbL film than for pristine samples.

**TABLE 1:** Kinetic parameters.

Diffusivity, D		$1.744\times 10^{-5}{\text{cm}}^{2}/\text{h}$
Partition rates		
UC-CFX solution, $\gamma_{L}$	Control	1.478
	LbL	3.928
UC-PBS solution, $\gamma_{D}$	Control	0.7688
	LbL	1.282

**FIGURE 7:**
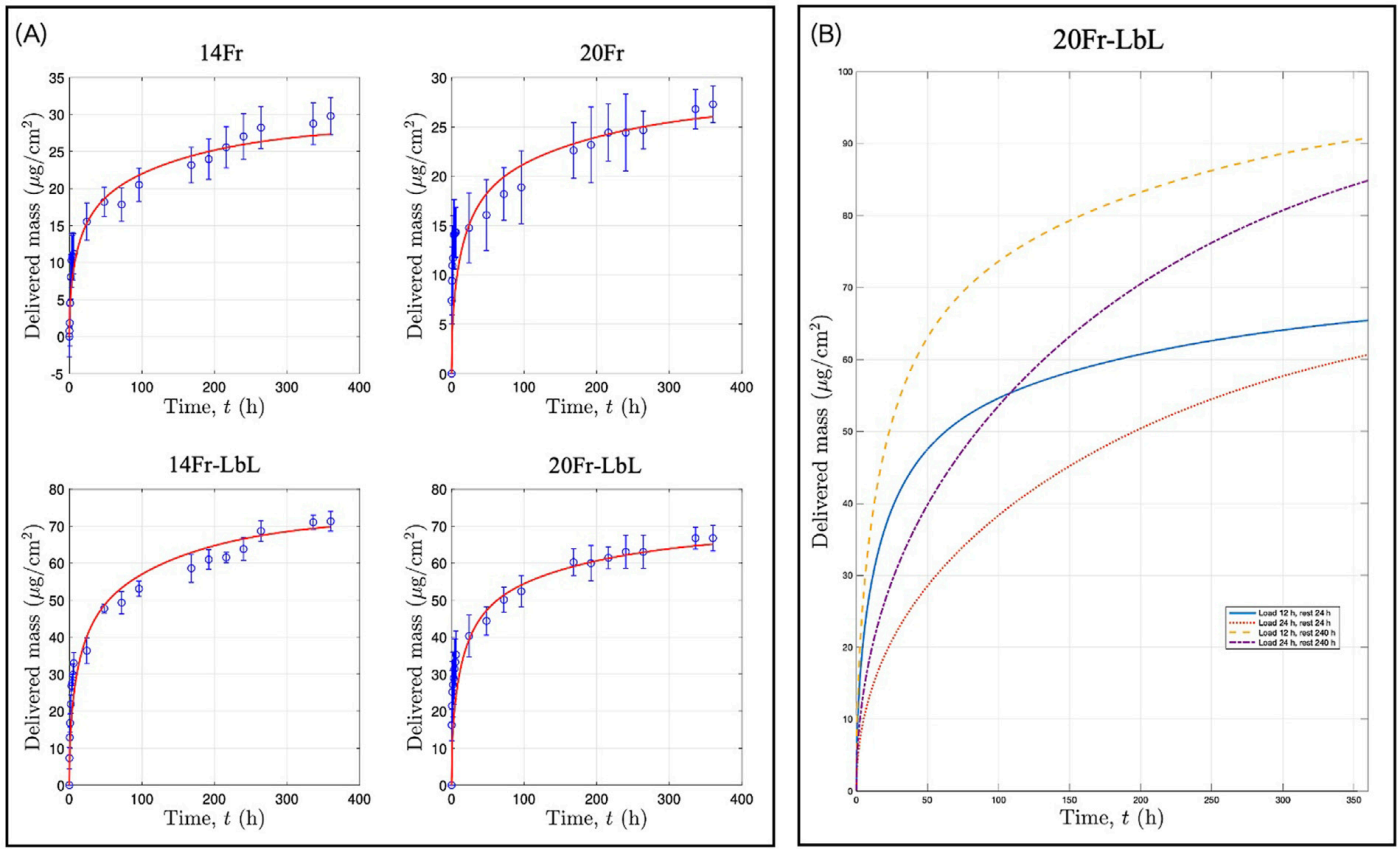
**(A)** CFX release profiles: experimental data and simulations. **(B)** Simulations of drug release profiles considering different drug loading and rest stage times.

Using the kinetic parameters from Table 1, several simulations were conducted. The Supplementary Figure S1A illustrates the CFX concentration inside the UCs immediately after the 12-h loading period. In all cases, both pristine (14Fr and 20Fr) and coated samples (14Fr-LbL and 20Fr-LbL) exhibited CFX accumulation at the border of the external wall of the UCs. However, after the 24-h rest stage preceding the drug delivery experiments (Supplementary Figure S1B), simulations showed how CFX continues to diffuse through the samples. With these insights, finally, we were able to simulate the experimental data of CFX delivery over the 15-day release period (Figure 7A). Additionally, owing to the characteristics of this mathematical model, we can predict how drug release profiles will differ based on varying drug loading and rest stage times (Supplementary Figure S1C; Figure 7B). Simulations indicated that extending the loading time may not necessarily result in higher CFX loading, as equilibrium is reached at the border of the external UCs wall. However, longer rest stage times enable more controlled release, as CFX becomes more homogeneously distributed throughout the samples.

**FIGURE 8:**
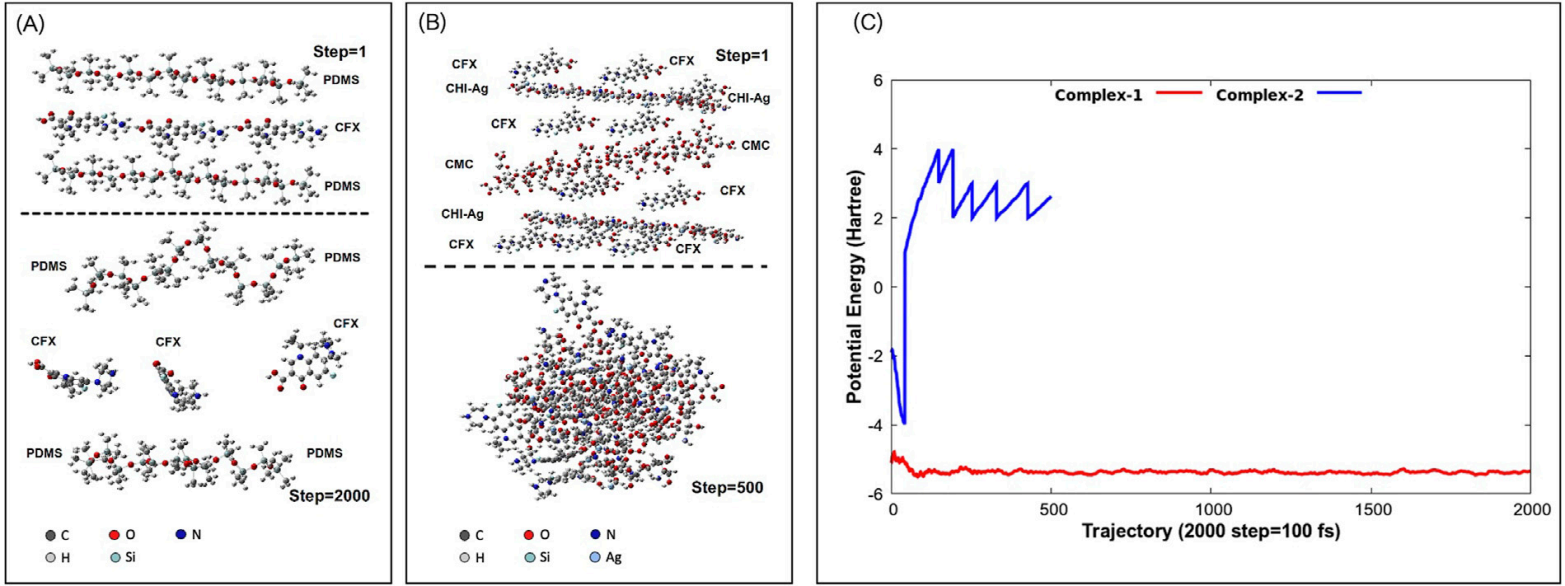
Molecular dynamics simulations: **(A)** Complex-1 [PDMS-CFX-PDMS] and **(B)** complex-2 [CFX-CHI-Ag-CFX-CMC-CFX-CHI-Ag-CFX]. 1 step = 0.05 fs. **(C)** Potential energy of simulated complexes.

Figure 8 presents the molecular dynamics simulations. Complex-1 (Figure 8A) shows a stable capacity for CFX loading within PDMS, as reflected by its consistent negative potential energy, approximately −5 $E_{h}$ (Figure 8C), which remains steady over time. In contrast, Complex-2 (Figure 8B), composed of micron-thick LbL layers, exhibits a greater capacity for CFX loading due to strong interactions between CFX and the CMC/CHI-Ag layers. This is illustrated by the notable contraction of the layers at step 500 (Figure 8B), in comparison to the loading of CFX into PDMS at step 2000 (Figure 8A). Furthermore, the observed increase in potential energy from −2 to 3 $E_{h}$ within the first femtoseconds illustrates the relatively lower stability of complex-2. This instability does not endanger the integrity of the structure, but rather, ensures proper biodegradation of the drug delivery system.

## 4 Conclusion

This study successfully developed a dual strategy to create an antibacterial surface on commercial Foley silicone urinary catheters (UCs) by combining the contact-killing effect with the controlled release of antimicrobial compounds. Specifically, UCs were coated with an antibacterial drug delivery system using a Layer-by-Layer (LbL) approach that incorporated carboxymethylcellulose (CMC) and chitosan-silver (CHI-Ag) complexes, loaded with ciprofloxacin (CFX) as the model drug. The very thin LbL coating significantly enhanced drug loading and release, achieving over twice the amount of CFX release compared to uncoated catheters. The antibacterial efficacy was markedly improved in the coated samples, particularly against *S. aureus* than *E. coli*. Additionally, a mathematical model based on Fick’s second law was proposed to simulate the experimental CFX release profiles, effectively predicting drug release kinetics by incorporating both drug loading and resting stages. Molecular dynamics simulations further confirmed strong compatibility between CFX and the LbL layers, although with relatively low stability. Despite the promising results, the study has certain limitations that should be addressed in future research. First, the antibacterial performance was evaluated only against planktonic forms of *E. coli* and *S. aureus*, without directly assessing biofilm formation or eradication on the catheter surface. Given the clinical importance of biofilms in CAUTIs, future studies should focus on quantifying biofilm inhibition and evaluating antimicrobial durability under physiologically dynamic conditions. Second, the release kinetics of silver from the layer-by-layer (LbL) coating were not investigated. Although silver was reduced to its elemental form ( ${\text{Ag}}^{0}$ ) and embedded within the matrix, its potential leaching over time could affect long-term antibacterial activity and should be examined in relation to biofilm suppression. Overall, these findings highlight the potential of this coating strategy to enhance the performance and safety of urinary catheters, particularly in reducing the incidence of catheter-associated urinary tract infections (CAUTIs).

## Data Availability

The raw data supporting the conclusions of this article will be made available by the authors, without undue reservation.
