# Supplementary material for: Drug delivery system based on an antibacterial layer-by-layer coating on urinary catheters: an experimental and simulation approach
Source: Front Bioeng Biotechnol. 2025 Aug 29;13:1614509. doi: 10.3389/fbioe.2025.1614509 (PMC12426174; doi:10.3389/fbioe.2025.1614509)
Supplement: Supplementary file 1 [file DataSheet1.pdf]

Drug Delivery System Based on an Antibacterial Layer-by-Layer Coating Urinary Catheters: An Experimental and Simulation

Nelson Naveas, Ruth Pulido, Francisco Javier Fernández-Alonso, Miguel Manso Silván, Leonardo Soriano, Carlos Torres-Ulloa, Karel Mena-Ulecia, Gonzalo Recio-Sánchez, Juan Paulo Garcia-Sandoval, Jacobo Hernandez-Montelongo

Supplementary Information

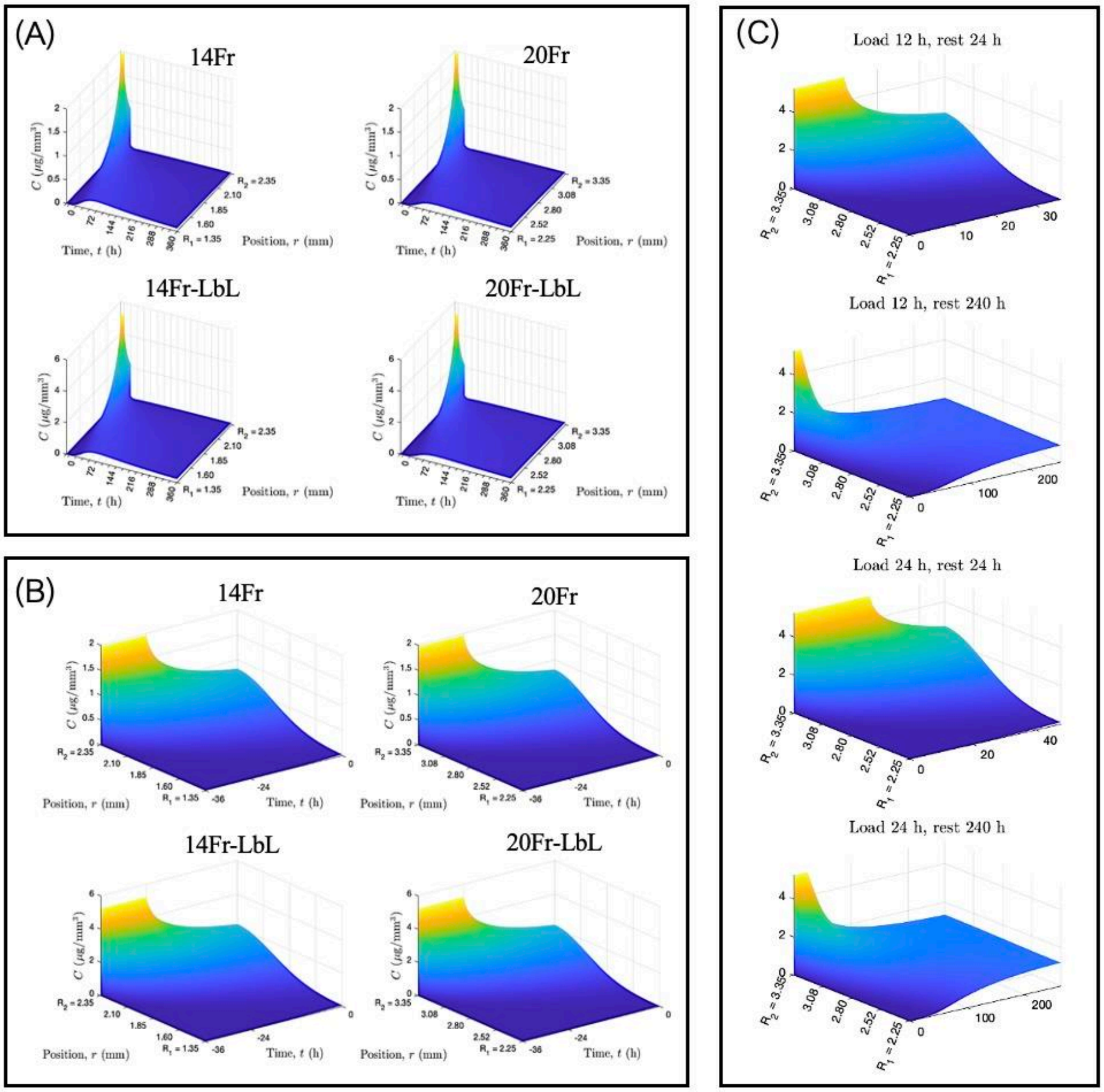

Figure S1. (A) CFX concentration profiles inside the UCs wall after 12 h of drug loading. (B) CFX concentration profiles inside UCs after 12 h drug loading and 24 h of rest stage. (C) CFX concentration profiles inside the UC wall after release considering different drug loading and rest stage times.
